# The prevalence and associated factors of alcohol use among pregnant women attending antenatal care at public hospitals Addis Ababa, Ethiopia, 2019

**DOI:** 10.1186/s12888-020-02747-1

**Published:** 2020-06-29

**Authors:** Getaneh Tesfaye, Demeke Demlew, Meseret G/tsadik, Fikreselam Habte, Gebeyaw Molla, Yohannes Kifle, Gebreslassie Gebreegziabher

**Affiliations:** 1 Department of Psychiatry, College of Health Sciences, Axum University, Aksum, Ethiopia; 2 College of Medicine and Health, Department of Psychiatry, University of Gondar, Gondar, Ethiopia; 3 St. Amanuel Specialized Mental Hospital, Addis Ababa, Ethiopia

**Keywords:** Alcohol use, Pregnant women, Public hospitals, Ethiopia

## Abstract

**Background:**

Alcohol use during pregnancy is a significant public health problem, ultimately affecting the neonatal offspring. Recent studies explore that no safe amount and safe time to drink alcohol during pregnancy. Even though drinking in pregnancy has a wide range of problems, a small number of scientific publications document on the magnitude of drinking alcohol during pregnancy in Sub-Saharan African countries including Ethiopia. The aim of this study was to assess prevalence and associated factors of alcohol use among pregnant women attending prenatal care at public hospitals, Addis Ababa, Ethiopia.

**Methods:**

Hospital based cross sectional study was employed from May 7 to June 6, 2019 at public hospitals, Addis Ababa. A total of 585 pregnant women participated in the study selected through systematic random sampling technique. Frequency of consumption was measured by using AUDIT. Frequency tables and graphs were used to describe the study variable. The association between variables analyzed with bi-variable and multivariable binary logistic regression. A statistical significance was declared at *p* value < 0.05 with 95% confidence interval.

**Result:**

A total of 585 participants were included in the study with the response rate of 98.6%. The study showed that the prevalence of alcohol use among pregnant women were 37.1% with (95% CI, 33.2–41). Factors like no formal education [AOR = 3.22, 95%CI, 1.72, 6.02], pre-pregnancy alcohol use [AOR = 3.16, 95%CI, 2.03, 4.91], partner alcohol use [AOR = 3.43, 95%CI, 2.21, 5.32], and poor social support [AOR = 3.16, 95%CI, 1.88, 5.31] were statistically associated with alcohol use during pregnancy.

**Conclusion:**

In this study the prevalence of alcohol use during pregnancy was high as compared to majority of other studies. This study observed that no formal education, pre-pregnancy alcohol use, partner alcohol use, and poor social support, were highly associated with alcohol use during pregnancy. Based on the findings of this study early management of alcohol use and problematic alcohol use is needed for pregnant women.

## Background

Alcohol is a psychoactive substance that can cause both acute and chronic changes in the brain [1]. Because of this drug with dependence-producing capability and leisure time activities many populations has been widely used it all over the world [2].

According to the WHO report in 2018, 3 million people lose their lives worldwide every year from harmful use of alcohol. The prevalence of Fetal Alcohol Spectrum Disorder (FASD) globally which is resulted from prenatal alcohol exposure among children and youth in the general population estimated to be 7.7 out of 1000 population [3].

Globally the magnitude of alcohol consumption during the index of pregnancy is estimated to be 10% [4]. In the USA the prevalence of alcohol use in pregnancy is 10.2% and women with productive age group reported a significantly higher prevalence of any alcohol use [5].

Countries in sub-Saharan African region alcohol exposure during pregnancy ranged from 2.2–87%. In this African region alcohol consumption in pregnancy is an increasing problem among pregnant women [6, 7].

In Ethiopia Both traditional and manufactured alcoholic drinks are used, the estimated alcohol content for different traditional alcoholic drinks is 2–4% for “*tella*” (traditional beer), 7–11% for “*tej*” (honey wine) and up to 45% for “*araqe*” (strong colorless liquor distilled from grain) [8]. According to 2015 national non-communicable diseases survey the overall prevalence of lifetime alcohol consumption in Ethiopia was 49.3% [9]. A study done in Bahir-Dar city showed 34% of respondent use alcohol during pregnancy at least once per week [10].

Alcohol use during pregnancy is a significant public health problem, ultimately affecting the neonatal offspring. A mother doesn’t have to be alcoholic for damaging effects of alcohol for the fetus. It means that no safe amount and safe time to drink alcohol during pregnancy period [11]. Alcohol use during pregnancy has been a well-known risk factor for adverse pregnancy outcomes. This including stillbirth [12], spontaneous abortion [13], premature birth [14], intrauterine growth retardation and low birth weight [15].

A Fetal Alcohol Spectrum Disorder (FASD) is an umbrella term for the wide range of adverse effects to the developing fetus when exposed to alcohol during pregnancy [16]. FASD includes, abnormal facial features called the philtrum, small head circumference, shorter than average height, low body weight, poor coordination, Attention deficit hyperactive disorder [17], memory problems, learning disabilities and school performance become decline, speech and language delays, intellectual disability or low IQ [18], poor reasoning and judgment skills [19], sleep and sucking problems as a baby, vision or hearing problems, problems with the heart, kidney, and bones [20–24]. The neuro-developmental impairments associated with FASD can later in life lead to substantial secondary disabilities such as academic failure, substance abuse, mental health problems, and an inability to live independently obtain and maintain employment [25].

In Canada the economic costs of FASD with productivity losses due to morbidity and premature mortality, cost of corrections and cost of health care was $1.8 billion in 2013 [26].

Though alcohol use and its burden in pregnant women is high a few scientific publications document on FASD and drinking in pregnancy in Sub-Saharan African countries [27].

Despite having devastating physical, mental and social consequence of alcohol consumption during pregnancy period, specifically in Ethiopia, few studies have explored the prevalence and predictors of its use and still studies are minimal to inform programmers and policy-makers. Therefore, the current study was conducted to assess the prevalence and associated factors of alcohol use in pregnant women.

## Methods

### Study design and period

Hospital based cross sectional study was employed from May 7 to June 6, 2019.

### Study area

The study area was Addis Ababa, the capital and largest city of Ethiopia. It is the seat of the Ethiopian federal government. It was founded by Emperor Menellik II in the late nineteenth century. In Addis Ababa, there are 12 public hospitals providing health services of medical management, surgical intervention, obstetric and gynecological management, antenatal care, pediatric, orthopedic, psychiatric and other essential service for a large number of people. From those with 12 hospitals according to high antenatal care service follow up five hospitals were selected. This includes St. Paul hospital, Zewditu memorial hospital, Yekatit 12 hospital, Minelik hospital and Ras desta hospital.

### Source population

All pregnant women who had antenatal care visit in the public hospitals of Addis Ababa.

### Study population

Pregnant women who were visiting antenatal care clinic at five selected public hospitals during study period.

### Sample size determination

Sample size was determined by using Single population proportion formula. By taking from proportion of Alcohol use in pregnancy 34% a study which was done in Bahir Dar, Ethiopia, with 4% margin of error, 95% CI and 10% non-response rate.

$$ n={Z_{\frac{\alpha }{2}}}^2\ast \frac{p\left(1-p\right)}{d^2}\kern1em n={1.96}^2\frac{0.34\left(1-0.34\right)}{0.04^2}\kern1em n=539 $$

Adding 10% non-response rate gives us a final sample size of **593.**

Where,

*n = Minimum sample size required for the study.*

*Z = Standard normal distribution (Z = 1.96) with confidence interval of 95% and ⍺ = 0.05.*

*P = Proportion of Alcohol use in pregnancy 34% a study which is done in Bahir Dar, Ethiopia.*

*d = Absolute precision or tolerable margin of error (d) = 4% = 0.04.*

Concerning the sampling technique which was employed in the study was Systematic random sampling. Study population come from selected five public hospitals; this include, St. Paul’s Hospital Millennium Medical College, Zewditu memorial hospital, yekatit 12 hospital, Minilik hospital and Ras desta hospital. Study population was selected proportionally, from each hospital.

Systematic random sampling was used to select study subjects from each hospital. The interval size (k) was calculated using the following formula.

$$ {\displaystyle \begin{array}{l}k=\frac{N}{n}\\ {}{k}_1=\frac{900}{121}=7.4\kern1em {k}_2=\frac{1500}{202}=7.4\kern1em {k}_3=\frac{300}{40}=7.5\kern1em {k}_4=\frac{500}{67}=7.4\kern1em {k}_5=\frac{1200}{162}=7.4\\ {}k\approx 7\end{array}} $$

Therefore, the interval size for each hospital was 7. So that every seven persons was selected from the study population.

Where *N*- Monthly population of selected hospitals.

*n*- Sample size of each hospital.

## Measures

### Measures for the dependent variable (alcohol use during pregnancy)

Respondents who answered “Yes” to the question “Have you ever consumed alcohol during your current pregnancy?” had alcohol use in pregnancy.

Alcohol Use Disorders Identification Test (AUDIT) is a 10-item alcohol screening instrument was used to measure the frequency of consumption and alcohol use disorder. It was developed by the World Health Organization and has been found effective in identifying subjects with a drinking problem such as hazardous drinking harmful drinking, and alcohol dependence (sensitivity, 94.1%; specificity, 91.7%). AUDIT was originally designed as an instrument for use in primary care settings; several recent studies have validated it in other health care and community contexts including pregnant women. The first three questions [1–3] explore quantity and frequency of alcohol consumption, the second three questions [4–6] explore signs of alcohol dependency and the last four questions [7–10] explore alcohol-related problems (harmful alcohol use) [28]. Response options for each item range from 0 to 4, resulting in a total possible score of 40.A total score of 1–7 indicates social drinking a score of 8–15 indicates “hazardous drinking” a score of 16–19 indicate “harmful drinking” and a score of 20 or above indicate probable alcohol dependence [28]. In this study we adapted the reference time of AUDIT the past 12 month to the time of pregnancy.

### Measures for the predictor variables

#### Socio-demographic characteristics

Collected by semi-structured socio demographic questionnaires, obstetric factor also was collected by semi-structured questionnaires, and substance related factors was collected by substance related questions.

#### Social support

Oslo-3 item social support scale, it is 3 item questionnaires, commonly used to assess social support and it has been used in several studies, the sum score scale ranging from 3 to 14, which has 3 categories: poor support 3–8, moderate support 9–11 and strong support 12–14 [29].

#### Other substance use

According to Alcohol, Smoking and Substance Involvement Screening Test (ASSIST).

**Current use**: using at least one of a specific substance for non-medical purpose within the last 3 months (khat, tobacco, others).

**Ever use of substance**: using at least one of any specific substance for non-medical purpose at least once in life time (khat, tobacco, others) [30].

#### Psychological distress

was measured using Kessler Psychological Distress Scale, 20–24 are likely to have a mild mental distress, score 25–29 are likely to have moderate mental distress and score 30 and over are likely to have a severe mental distress [31].

#### Intimate partner violence

Measured using HITS screening tool. During the HITS assessment, a provider asks a pregnant the following: How often does your partner physically Hurt you, Insult or talk down to you, threaten you with harm, and Scream or curse at you? Each category is graded on a scale of 1 (never) to 5 (frequently) and a sum of all the categories is generated. A total score of 10 is suggestive of IPV [32].

### Data collection procedures

Data was collected using face to face interview with questionnaire. The data was collected by 5 BSc. Female nurses, and supervised by two psychiatric nurses. Consequently, the entire data collection process had seven members. The nurses were employees of the hospitals. Accordingly, for each selected five hospitals there was one data collector and the supervisors were supervising them on each day. Training was provided to data collectors and supervisors for 2 days on methodology, ethical issue and how to administer questionnaires.

### Data quality control

The entire questionnaire was translated into local languages Amharic then it was translated back to English by an independent person to check for consistency and understandability of the tool. The questionnaire was pretested 1 week prior to the actual data collection on 5% of sample size at Addis Ketema Felege Meles health center in antenatal clinic and the questionnaire was checked for its clarity, simplicity, and understandability and items of questions was modified accordingly. Data collectors were supervised daily and the filled questionnaire was checked daily by the supervisors.

### Data processing and analysis

The collected data was checked visually for its completeness and the response was coded and entered into the computer using EPI data version 3.1, and then cleaned. The cleaned data was exported to SPSS Version 20 for analysis. Then the results were summarized and presented by tables, and charts. Furthermore, Percentage, frequency and mean were calculated. Firstly, bi-variate binary logistic regression was performed to screen determinant factors of the outcome variable. Secondly, those predictor variables which were significantly associated with outcome variable with a *p*-value< 0.25 in the bi-variable logistic regression analysis were entered into the multivariate logistic regression model for controlling the possible effect of confounders. The strength of the associated factors was presented by odds ratio with 95% confidence interval. The variables which have a statistical association were identified on the basis of *p*-values ≤0.05. The model fitness for multivariate binary logistic regression was checked by using Hosmer and Lemeshow test.

### Ethical consideration

Ethical clearance was obtained from ethical review committee office of Amanuel Mental Specialized Hospital, University of Gondar, College of medicine and health science, Addis Ababa regional health bureau and St. Paul’s hospital Millennium medical college. Written informed consent was secured from each participant during study period. Participants’ right to refuse the participation was kept. For some clinical outcome patients was linked to psychiatry support as necessary and for the participants who were found problematic alcohol users, psychological distress positive during the study, communication to nearby psychiatric clinic was done in order to have further assessment on their condition. Confidentiality of respondents was maintained.

## Result

### Socio-demographic characteristics of the respondents

A total of 585 participants were included in the study with the response rate of 98.6%. The mean age (±SD) of the respondents was 27.31(±4.5), with age ranging from 18 to 43 years. Among the respondents, the highest age was in a range of 25–29 years 272(46.5%). Of the total participants about400 (68.4%) were orthodox religion follower. The majority of the participants were married 542 (92.6%). The educational status of participants indicated that about 167(28.5%) of them attended secondary level of school and most of them were housewives 378(64.6%). Large numbers of respondents were from urban 548(93.7%). (Table 1).

**Table 1:** Distribution of participants by socio-demographic factors visiting antenatal clinics at public hospitals Addis Ababa, Ethiopia, 2019 (*n* = 585)

Variable	Frequency(*N* = 585)	Percent (%)
Age		
18–19	10	1.7
20–24	139	2.38
25–29	272	46.5
30–34	120	20.5
35and above	44	7.5
Religion		
Orthodox	400	68.4
Muslim	109	18.6
Protestant	74	12.6
Catholic	2	0.3
Maternal educational status		
No formal education	146	25
Primary education	114	19.5
Secondary education	167	28.5
Preparatory	30	5.1
College and above	128	21.9
Occupational status		
Farming	16	2.7
Merchant/private	90	15.4
Government employee	101	17.3
House wife	378	64.6
Marital status		
Married	542	92.6
Not married	39	6.7
Divorced	4	0.7
Residence		
Urban	548	93.7
Rural	37	6.3

### Obstetric characteristics of the respondents

During the study period, 170(29.1%), 243(41.5%) and 172(29.4%) subjects were in the first, second and third trimester of pregnancy, respectively. Thirty-five percent of the study subjects were multiparous and 491(83.9%) of the pregnancies were planned. Besides, history of abortion was experienced by (107)18.3% of respondents. (Table 2).

**Table 2:** Obstetric factor of the participant visiting antenatal clinics at public hospitals Addis Ababa, Ethiopia, 2019 (*n* = 585)

Variable	Frequency(*N* = 585)	Percent (%)
Gestational age		
First trimester	170	29.1
Second trimester	243	41.5
Third trimester	172	29.4
Parity		
Null para	156	26.7
Has one child	155	26.5
Has two children	204	34.9
Has three and above children	70	12
Pregnancy		
Planned	491	83.9
Unplanned	94	16.1
History of abortion		
Yes	107	18.3
No	478	81.7

### Maternal psychosocial characteristics

The current study revealed poor social support accounts 188(32.1%), maternal with psychological distress was 161(27.5%) and intimate partner violence reported by the respondents include 39(6.7%).

### Prevalence of alcohol use among pregnant women

Alcohol use during pregnancy was reported by about 217(37.1%) with (95% CI, 33.2–41). Among this the majority use alcohol during the second trimester 100(46.1%) and most of them were married 203(93.5%) and they had history of pre-pregnancy alcohol use 113(52.1%).

According to alcohol use disorder identification test (AUDIT), most of them 124(57.1%) pregnant women use alcohol monthly or less. Regarding amount of drinking majority of pregnant women 114(52.5%) drink alcohol up to one to two drinks during the time of drinking.

Alcohol use disorder (AUDIT score 8 or more) among pregnant was 8(3.7%). Hazardous and harmful drinkers were 6(2.8%) and 2(0.9%) respectively. Of users heavy episodic drinking (six or more units on a single occasion) at least monthly or more frequently include 86(39.6%).

The most frequent type of alcohol they drink is beer 95(43.8%) followed by “Tella”58(26.7%) and wine 52(24%). The reason why most pregnant women drink alcohol is to relaxation 81(37.3%), socialization 58(26.7%) and peer pressure accounts 41(18.9%). (Table 3).

**Table 3:** Distribution of alcohol use among pregnant women visiting antenatal clinic at public hospitals Addis Ababa Ethiopia. 2019

Variables	Frequency	Percent
Alcohol use during pregnancy *N* = 585		
Yes	217	37.1
No	368	62.9
Frequency of drinking *N* = (217)		
Monthly or less	124	57.1
2–4 times a month	67	30.9
2–3 times a week	20	9.2
4 or more times a week	6	2.8
Amount of drinking *N* = (217)		
1–2 drinks	114	52.5
3–4 >>	52	24
5–6 >>	44	20.3
7–9 >>	7	3.2
AUDIT score 8 or more (alcohol use disorder) *N* = (217)	8	3.7
Hazardous drinking (AUDIT score of 8–15)	6	2.8
Harmful drinking (AUDIT score of 16–19)	2	0.9
Dependency (AUDIT score 20 and above)	–	–
Heavy episodic drinking (six or more units on a single occasion) *N* = (217)	86	39.6
Reason of alcohol use during pregnancy *N* = (217)		
For relaxation	81	37.3
Socialization	58	26.7
Peer pressure	41	18.9
To get relief from stress	37	17.1
Type of alcohol they use *N* = (217)		
Beer	95	43.8
Tella	58	26.7
Wine	52	24
Draft	8	3.7
Tej	3	1.4
Araque	1	0.5

### Other substance use history (ever and current use)

With the exception of alcohol about 101(17.2%) of the respondents had history of substance use with in the past 3 months. Majorities of them reported that, they were chewing khat 61(10.4%). Among participants, 33(5.6%) of the respondents were smoking cigarette with in the past 3 months. (Table 4).

**Table 4:** Substance use history with the exception of alcohol use among participants visiting antenatal clinic at public hospitals Addis Ababa Ethiopia. 2019

Variables	Frequency	Percent (%)
**Ever cigarette use**		
Yes	119	20.3
No	466	79.7
**Ever khat use**		
Yes	171	29.2
No	414	70.8
**Current cigarette use**		
Yes	33	5.6
No	552	94.4
**Current khat use**		
Yes	61	10.4
No	524	89.6
**Other substance use** (ever)		
Yes	13	2.2
NO	572	97.8
**Other substance use** (current)		
Yes	7	1.2
NO	578	98.8

❖ N.B. Other substance use is (Cannabis, Amphetamine, opioids)

### Factors associated with alcohol use among pregnant women

In bi-variable binary logistic analysis variables; no formal education, living in urban place, multi parity, no pregnancy plan, pre-pregnancy alcohol use, partner alcohol use, psychological distress, having intimate partner violence, poor social support, current khat use were found to have *p*-value less than 0.25. Those variables fulfilled minimum requirement for further multivariate binary logistic regression.

From multivariate binary logistic regression only variables no formal education, pre-pregnancy alcohol use, partner alcohol use, and poor social support, were statistically associated with alcohol use during pregnancy at p-value less than 0.05.

The odds of having alcohol use during pregnancy among respondents with no formal education was 3 times higher as compared to those with having educational level of college and above [AOR = 3.22, 95%CI, 1.72, 6.02].

Women with whose partner alcohol use were 3.4 times more likely to drink alcohol during pregnancy than women with a partner not users of alcohol [AOR = 3.43, 95%CI, 2.21, 5.32].

Pregnant women with poor social support were 3.16 times more likely to use alcohol during pregnancy as compared to those with strong social support [AOR = 3.16, 95%CI, 1.88, 5.31].

*The odds of having alcohol use during pregnancy among respondents with pre- pregnancy alcohol use was 3 times higher as compared to those with none pre-pregnancy alcohol users [AOR= 3.16, 95%CI, 2.03, 4.91]. (*Table 5*)*

**Table 5:** Bi-variable and multivariable binary logistic regression analysis showing association between factors and alcohol use among pregnant women visiting antenatal clinics at public hospitals Addis Ababa Ethiopia, 2019(*N* = 585)

Explanatory variables	Alcohol use		COR, (95% CI)	AOR, (95%CI)
	Yes	No		
Educational status				
Have no formal education	80	66	3.48 (2.08, 5.82)	3.22 (1.72, 6.02) **
Primary	42	72	1.67 (0.97, 2.90)	1.08 (0.57, 2.07)
Secondary	57	110	1.49 (0.89, 2.48)	1.44 (0.80, 2.60)
Preparatory	5	25	0.57 (0.20, 1.62)	0.49 (0.14, 1.70)
College and above	33	95	1	1
Residence				
Urban	207	341	1.63 (0.77, 3.45)	1.92 (0.75, 4.93)
Rural	10	27	1	1
Parity				
Nulliparous	51	105	1	1
Primipara	59	96	1.26 (0.79, 2.01)	1.12 (0.64, 1.96)
Multipara	107	167	1.31 (0.87, 1.99)	1.37 (0.83, 2.26)
Pregnancy plan				
Yes	171	320	1	1
No	46	48	1.79 (1.14, 2.79)	1.14 (0.66, 1.98)
Pre-pregnancy alcohol use				
Yes	113	75	4.24 (2.93, 6.13)	3.16 (2.03, 4.91) **
No	104	293	1	1
Partner alcohol use				
Yes	120	100	3.31 (2.33, 4.71)	3.43 (2.21, 5.32) **
No	97	268	1	1
Intimate partner violence				
Yes	20	19	1.86 (0.97, 3.57)	1.62 (0.72, 3.63)
No	197	349	1	1
Social support				
Poor	120	68	5.19 (3.38, 7.97)	3.16 (1.88, 5.31) **
Moderate	43	141	0.89 (0.56, 1.42)	0.66 (0.39, 1.11)
Strong	54	159	1	1
Current khat use				
Yes	27	34	1.39 (0.81, 2.38)	1.23 (0.61, 2.45)
No	190	334	1	1
Psychological distress				
Yes	81	80	2.14 (1.48, 3.10)	1.56 (0.97, 2.53)
No	136	288	1	1

NB. 1.00 reference **p-value less than 0.001

The variables in AOR were identified on the basis of *p*-values ≤0.05

Chi square = 8.71, df = 8, Hosmer lemshow test = 0.367

## Discussion

This study assessed the prevalence of maternal alcohol consumption during pregnancy, as well as predictors of this behavior in Addis Ababa public hospitals.

The prevalence of alcohol use during pregnancy in this study was 37.1% with (95% CI, 33.2–41). The finding of the current study was similar with studies carried out in brazil 32.4% [33], Ghana 37.6% [34], Geneva 36.3% [35] and another study in Ethiopia, Bahr-Dar city [10] which was 34%. However, the current study was less than the study was done in Ukraine 46.3% [36],59% in Nigeria [37] and another study in Ghana 48% [38]. The possible reason for this difference might be variation in study design which was the prospective cohort study that was conducted in 2 regions of Ukraine. It might be also due to a difference in a study setting in which samples are taken, women in James Town, a community-based study done in Ghana [38].

On the other hand, the finding of this study was higher than studies done in Sweden 6% [39], Canada 10% [40], Thailand 5.8% [41], Australia 29% [42]. This variation might be due to only pregnancy week 18 or more was the study participants in Sweden. Study design and sampling technique which was multi-stage stratified random sampling and a list assisted random digit dialing sampling technique were used by telephone in Australia [42]. Socio-cultural and study design variation might be another possible reason for the discrepancy, in which registry-based survey was conducted in Canada [40]. An instrument used might be another reason for this variation; alcohol use was assessed by using ASSIST-lite and AUDIT-C in Thailand and Sweden respectively. AUDIT-C assesses the risk of drinking and ASSIST instrument only assess alcohol users within 3-months duration.

Multivariate logistic regression revealed that no formal education, pre-pregnancy alcohol use, partner alcohol use, and poor social support had a statistically significant association with alcohol use during pregnancy.

In this study, no formal education level was associated with alcohol use during pregnancy. This observation is consistent with results found in Ukraine [36], Spain [43] and Uganda [44], maternal educational status is a protective and the factor that influence alcohol cessation during pregnancy. The possible justification for this association could be women with higher education would be aware of the risk of drinking during pregnancy and they might have behavioral change through learning. However, in countries such as Japan [35], Tanzania [45] higher education was among the factor that influence alcohol use during pregnancy. High socio-economic and different cultural status in Japan.

In the present study pre-pregnancy alcohol use was predictive of alcohol use during pregnancy. This finding is similar with studies in Sweden [39], Tanzania [45], Australia [46]. This association might be alcohol produces a physiological effect such as the strong desire to consume despite knowing the outcomes thus when an individual has drinking tendency this becomes a habit that is difficult to break.

With the current study alcohol use for pregnant women who had poor social support were three times higher than those who had strong social support. This finding is similar to a study done in Sweden [39]. Having someone around and good social support can lead to reducing drinking by helping individual positively cope with stress and mitigating depression. Besides, emotional support such as encouragement for abstinence can have an important role not to continue drinking.

Women with whose partner alcohol use were three times more likely to drink alcohol during pregnancy than women with a partner not users of alcohol. This is supported by the study conducted in Tanzania, Uganda and other study done in Ethiopia, Bahar-Dar [10, 44, 45]. This might be because relatives play a role as an essential role model for an individual to decide to drink and sometimes one can be invited to drink alcohol and becomes difficult for her to resist.

## Conclusion and recommendations

In the current study, the prevalence of alcohol use during pregnancy was high as compared to the majority of other studies. This study observed that no formal education, pre-pregnancy alcohol use, partner alcohol use and poor social support were statistically significant with alcohol use during pregnancy. Alcohol-related health education program should be strengthened for those with no formal education that helps to empower pregnant women with knowledge, and strengthens family social support to combat alcohol use during pregnancy and its effect on the newborn and the pregnant mother itself. Alcohol use screenings and problematic alcohol use assessments for diagnosis of problems early and intervention should be carried out.

## Limitations

The limitation of this study might be recalling problem (recalling bias) of some behaviors. It also might face to social desirability bias which is the tendency of the participants to answer the questions according to socially accepted manner and they may under report their alcohol consumption. Another limitation may arise due to difficulties with people’s perceptions of what constitutes ‘a drink’. Estimating quantity and frequency of alcohol consumed is difficult due to lack of understanding about alcohol units, and is dependent on glass size and drink strength.

## Data Availability

The datasets used and analyzed during the current study are not publically available due to ethical restriction and personal data protections but are available from the corresponding author on reasonable request.

## Abbreviations

AEP: Alcohol exposed pregnancy
AOR: Adjusted odds ratio
AUDIT: Alcohol use identification test
CI: Confidence interval
COR: Crude odds ratio
FAS: Fetal alcohol spectrum
FASD: Fetal alcohol spectrum disorder
IQ: Intelligent quotient
OR: Odds Ratio
SAUs: Standard alcohol units
T-ACE: Tolerance, annoyed, cut off, and eye opening
TWEAK: Tolerance, worry, eye opener, amnesia, kut/cut down
UK: United Kingdom
USA: United States of America
WHO: World health organization
