# Correction to: The prevalence and associated factors of alcohol use among pregnant women attending antenatal care at public hospitals Addis Ababa, Ethiopia, 2019

**DOI:** 10.1186/s12888-020-02876-7

**Published:** 2020-10-06

**Authors:** Getaneh Tesfaye, Demeke Demlew, Meseret G/tsadik, Fikreselam Habte, Gebeyaw Molla, Yohannes Kifle, Gebreslassie Gebreegziabhier

**Affiliations:** 1 Department of Psychiatry, College of Health Sciences, Axum University, Aksum, Ethiopia; 2 College of Medicine and Health, Department of Psychiatry, University of Gondar, Gondar, Ethiopia; 3 St. Amanuel Specialized Mental Hospital, Addis Ababa, Ethiopia

**Correction to: BMC Psychiatry 20, 337 (2020)**

**https://doi.org/10.1186/s12888-020-02747-1**

Following publication of the original article [1], the authors identified an error in the author name of Gebreslassie Gebreegziabhier.

1. Incorrect name: Gebreslassie Gebreegziabher
2. Correct name: Gebreslassie Gebreegziabhier

The author group has been updated above and the original article [1] has been corrected.
